# Effects of Environmental Factors on the Mechanical Properties of Palm Leaf Manuscripts: Natural Aging, Temperature, Relative Humidity, and Light Radiation

**DOI:** 10.3390/polym17233229

**Published:** 2025-12-04

**Authors:** Wenjie Zhang, Shan Wang, Hong Guo

**Affiliations:** 1 Key Laboratory of Archaeomaterials and Conservation, Ministry of Education, Institute of Cultural Heritage and History of Science & Technology, University of Science and Technology Beijing, Beijing 100083, China; d202310769@xs.ustb.edu.cn; 2 Chinese Academy of Cultural Heritage, Beijing 100029, China; cnicpbj@gmail.com

**Keywords:** *Corypha umbraculifera*, cellulose, mechanical properties, simulated aging experiment, preventive conservation

## Abstract

The mechanical properties of palm leaf manuscripts, a unique organic cultural heritage material, are strongly influenced by environmental conditions that directly determine their physical stability and long-term preservation. This study systematically examined the effects of natural and accelerated aging under different temperature, humidity, and light radiation conditions on the mechanical and chemical properties of palm leaf samples. Flexural strength and flexural modulus were measured to assess mechanical degradation, while FT-IR was employed to evaluate chemical structure changes. The results revealed that temperature had a pronounced effect on mechanical performance. At −20 °C, a temporary increase in flexural strength and modulus was observed due to the structural stabilization caused by frozen moisture, followed by a gradual decline attributed to ice crystal rupture and fiber damage. At 25 °C, degradation progressed steadily, while at 100 °C, the material underwent severe shrinkage and deformation, resulting in significant cracking and a subsequent sharp decline in its mechanical properties. Relative humidity also played a critical role: excessive dryness (10% RH) led to shrinkage and cracking, whereas high humidity (90% RH) caused microbial degradation and hydrolysis, both resulting in sharp declines in strength and stiffness. Samples aged at moderate humidity (50% RH) maintained superior mechanical stability. Light radiation further accelerated deterioration of mechanical properties, with UV exposure inducing the most significant loss due to photochemical reactions that disrupted lignin structures. FT-IR analysis confirmed that the degradation of cellulose and hemicelluloses was a significant cause of mechanical weakening. Overall, extreme environmental conditions accelerated both physical and chemical deterioration. Conversely, moderate and stable environments (around 25 °C, 50% RH, and limited light exposure) were found to be optimal for preserving the mechanical and structural stability of palm leaf manuscripts. These findings provide valuable guidance for the long-term conservation and environmental control of ancient organic manuscripts.

## 1. Introduction

Before the widespread use of paper, palm leaf manuscripts served as one of the most important writing media across South and Southeast Asia. Made from processed palm leaves, these manuscripts carry rich historical and cultural information, encompassing knowledge in religion, literature, philosophy, art, and science, and play a vital role in the transmission of Buddhist culture and regional thought exchange [1,2,3]. Owing to their significant historical and artistic value, palm leaf manuscripts are recognized as precious organic cultural heritage, and their restoration and preventive conservation have become major concerns in cultural heritage preservation.

In recent decades, research on palm leaf manuscripts has primarily focused on the analysis of their raw materials [4,5,6], production techniques [7,8], and digital documentation, cataloging, and textual deciphering [9,10,11]. Some researchers have also evaluated their preservation state and deterioration characteristics [12,13], and, on this basis, implemented conservation and restoration measures that have achieved positive outcomes [14,15]. These studies have laid a solid foundation for the scientific understanding and preservation practice of palm leaf manuscripts.

However, studies on the effects of environmental factors on the aging behavior of palm leaf manuscripts remain limited, particularly the lack of quantitative investigations that have been conducted into the variations in their mechanical properties under different environmental conditions. The mechanical performance of palm leaf manuscripts is a key indicator of their structural stability and restorability, directly influencing their preservation, restoration, and utilization, and thus, is critical to the protection and sustainable use of this unique cultural heritage. Similar to other lignocellulosic materials such as paper and wood, palm leaves are mainly composed of cellulose, hemicelluloses, and lignin [1,4,5]. A study confirmed that the content of cellulose, hemicellulose and lignin in palm leaves processed by traditional techniques such as boiling and sun-drying is approximately 53.6%, 14.4% and 10.8%, respectively [16]. This chemical composition endows palm-leaf manuscripts with advantages similar to those of paper and other lignocellulosic materials, such as renewability, high mechanical strength, and low cost. However, it also renders them highly sensitive to environmental factors like temperature, humidity, and light radiation, thereby readily altering their mechanical properties.

Previous studies on the accelerated aging of lignocellulosic materials such as paper and wood have yielded extensive results, which can provide valuable references for research on palm leaf manuscripts. For instance, some studies have confirmed that high-temperature environments, such as 100 °C, 130 °C, or higher, can alter the molecular structure of cellulose and hemicellulose, thereby affecting the flexibility and strength of paper or wood [17,18]. Excessively dry (30% RH) or (75% RH) relative humidity conditions may lead to desorption or moisture absorption in lignocellulosic materials, resulting in dimensional changes and internal stress that compromise mechanical stability [19,20]. Additionally, optical radiation such as ultraviolet light can initiate photochemical reactions, weaken fiber structures, and accelerate degradation [21,22]. The long-term influence of these environmental factors can cause varying degrees of deterioration in the mechanical, physical, and chemical properties of palm leaf manuscripts, ultimately affecting their preservation lifespan [23]. Therefore, systematically investigating the effects of environmental factors on the mechanical properties of palm leaf manuscripts is of great importance for revealing their aging mechanisms, assessing suitable conservation environments, and developing scientifically grounded preventive conservation strategies.

Unlike traditional mechanical testing instruments such as universal testing machines, which require relatively large sample dimensions, thermomechanical analysis (TMA) offers the advantages of minimal sample consumption, high precision, and excellent reproducibility. Our previous research utilized this technological achievement to test the short-term and long-term effects of relative humidity on mechanical properties of palm leaf manuscripts. It also demonstrated that TMA is particularly suitable for fragile and valuable organic heritage materials such as palm leaf manuscripts [24]. Based on this research, we carried out longer-term aging experiments and attempted to introduce different environmental factors, such as temperature and light radiation, to further examine the effects of these factors on the mechanical properties of palm leaf manuscripts.

In this study, palm leaf samples from different years of natural preservation were selected as the research objects. By combining simulated aging experiments with mechanical testing, microstructural observation, and Fourier-transform infrared spectroscopy (FT-IR) analysis, the mechanical behavior of palm leaf manuscripts under various environmental conditions was systematically investigated. This study aims to elucidate the effects of different environmental factors on the mechanical performance of palm leaf manuscripts, thereby enhancing understanding of the environmental influences on their longevity and providing essential data to support studies on deterioration mechanisms and preventive conservation strategies.

## 2. Materials and Methods

### 2.1. Experimental Materials

The experimental samples in this study were raw palm leaf manuscripts from Yunnan Province, China. These samples were produced following the traditional palm-leaf manuscript-making process, which has been recognized as part of China’s national intangible cultural heritage. The preparation involved boiling, washing, air drying, trimming, and flattening the leaves from the talipot palm (*Corypha umbraculifera* L.) tree [7], resulting in the creation of the samples used in this study. The size of each sample is approximately 45 cm × 5 cm. The samples were stored in a cool and dry repository (temperature of 16~22 °C, relative humidity of 30% RH~60% RH, avoid direct sunlight) for different durations—0 years (newly prepared), 2 years, 5 years, 8 years, and 12 years—to investigate the effects of natural aging on their properties.

**Figure 1:**
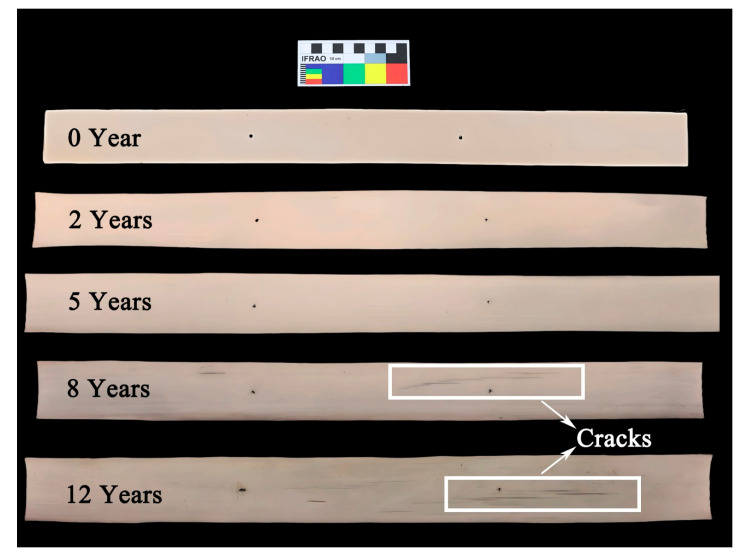
Photographs of samples after storage for 0, 2, 5, 8, and 12 years.

This study primarily examines how various environmental factors influence the mechanical properties of palm leaf manuscripts. Therefore, the raw palm leaf manuscript samples were not subjected to subsequent treatments, such as writing or coloring, in order to avoid any influence from pigments or binding materials on the study results. As shown in Figure 1, visible cracks have appeared on the surfaces of samples stored for longer periods, particularly those aged for 8 and 12 years.

### 2.2. Simulated Aging Experiment

#### 2.2.1. Temperature Aging Experiment

A portion of the samples stored for 0 years (newly prepared) was divided into three groups and placed in an environmental testing chamber (GSH-64, Espec, Osaka, Japan), each subjected to different aging conditions (temperature set to −20 °C, 25 °C and 100 °C, and relative humidity set to 50% RH for −20 °C and 25 °C, while no humidity control was applied at 100 °C). A portion of the samples was removed for flexural strength testing every 50 days. After 200 days of aging, the samples were removed and analyzed for morphological changes using a super-depth microscope (VHX-6000, KEYENCE, Osaka, Japan). Additionally, some samples were taken for infrared spectroscopy testing.

#### 2.2.2. Humidity Aging Experiment

A portion of the samples stored for 0 years (newly prepared) was divided into three groups and placed in an environmental testing chamber (GSH-64, Espec, Osaka, Japan), each subjected to different aging conditions (temperature set to 25 °C, and relative humidity set to 10% RH, 50% RH and 90% RH). A portion of the samples was removed for flexural strength testing every 50 days. After 200 days of aging, the samples were removed and analyzed for morphological changes using a super-depth microscope (VHX-6000, KEYENCE, Osaka, Japan). Additionally, some samples were taken for infrared spectroscopy testing.

#### 2.2.3. Light Radiation Aging Experiment

A portion of the samples stored for 0 years (newly prepared) was divided into three groups and placed in a UV aging chamber (UV Test, Atlas, Mount Prospect, IL, USA) and an environmental test chamber (GSH-64, Espec, Osaka, Japan) under different aging conditions to simulate various types of light radiation:

- Ultraviolet (UV) radiation: Conducted in the UV aging chamber, with a radiation wavelength of 340 nm, an irradiance of 1.51 W/m^2^, and a temperature of 55 °C.
- Visible light: Performed in the environmental test chamber equipped with a ring-shaped white LED light source (64 LED Lights, Yike, Shenzhen, China). The radiation wavelength ranged from 400 to 645 nm, with an output power of 10 W, at 25 °C and 50% RH.
- Infrared (IR) radiation: Performed in the environmental test chamber equipped with a ring-shaped infrared LED light source (HS-RD0, Hongshuo Technology, Hangzhou, China). The radiation wavelength ranged from 800 to 1750 nm, with an output power of 10 W, at 25 °C and 50% RH.

A portion of the samples was removed for flexural strength testing every 50 days. After 200 days of aging, the samples were removed and analyzed for morphological changes using a super-depth microscope (VHX-6000, KEYENCE, Osaka, Japan). Additionally, some samples were taken for infrared spectroscopy testing.

### 2.3. Flexural Strength Testing

Unlike traditional mechanical testing instruments such as universal testing machines, which require relatively large sample dimensions, TMA offers the advantages of minimal sample consumption, high precision, and excellent reproducibility.

The flexural strength of the samples was assessed using a thermomechanical analyzer (TMA 7100, Hitachi, Tokyo, Japan) equipped with quartz probes and components. The test was conducted with an initial load of 0.1 mN and a loading rate of 30 mN/min, continuing until the samples fractured to obtain the corresponding test curves. Measurements were taken every 50 days, with both the longitudinal and transverse directions of each sample tested, and each group comprising 20 samples.

The flexural strength (σ) and flexural modulus (E) of the samples were calculated using Formulas (1) and (2), respectively:

(1) $$\sigma =\frac{3FL}{2bd^{2}}$$

(2) $$E=\frac{FL^{3}}{4\delta bd^{3}}$$

where σ (MPa) and E(MPa) are the flexural strength and flexural modulus of the sample, respectively; F (N) is the maximum load at the point of sample fracture; L (mm) is the span between the supports on the TMA flexural strength testing platform, fixed at 5 mm; b (mm) and *d* (mm) are the width and thickness of the sample, respectively, measured using a super-depth microscope (VHX-6000, KEYENCE, Osaka, Japan); δ (mm) is the maximum displacement of the sample at the point of fracture.

### 2.4. FT-IR Test

The aged samples were finely ground and passed through a 200-mesh sieve. They were then mixed with KBr (spectrally pure, Macklin, Shanghai, China) at a mass ratio of 1:100 and pressed into pellets. The chemical structure of the samples was characterized using Fourier transform infrared spectroscopy (Nicolet™ iS™5, Thermo Scientific, Waltham, MA, USA) to compare the differences in the characteristic functional groups before and after aging.

In order to further characterize the changes to the chemical structure of the samples, the infrared spectra of the samples were analyzed semi-quantitatively, using the peak intensity method. The absorption peaks at 1730 cm^−1^ (stretching vibration of the carbonyl group of hemicelluloses) and 1460 cm^−1^ (stretching vibration of the methylene group of hemicelluloses) were selected as the characteristic peaks of hemicelluloses, and the absorption peak at 1505 cm^−1^ (backbone vibration of butyl propane in butyl lignin) was chosen to represent lignin, and the absorption peaks at 1370 cm^−1^ (bending vibration of the methyl group of cellulose) and 1050 cm^−1^ (bending vibration of the glycosidic bond of cellulose) were chosen to represent cellulose [25,26,27]. The intensity values of the infrared spectral peaks were measured after baseline correction using software (OMNIC 9.2, Thermo Scientific, Waltham, MA, USA).

### 2.5. Statistical Analysis

To compare the differences among different treatment groups, statistical analyses were conducted using SPSS 26.0 (IBM Corp., Armonk, NY, USA). Normality and homogeneity of variance were first tested to ensure the data met the assumptions of one-way analysis of variance (ANOVA). When a significant overall difference was detected (*p* < 0.05), Tukey’s Honestly Significant Difference (HSD) post hoc test was performed to determine pairwise differences among groups. The results of the significance analysis were visualized using the Compact Letter Display (CLD) method: different letters indicate significant differences between groups (*p* < 0.05), while the same letter denotes no significant difference. All data in the figures are presented as mean ± standard deviation (Mean ± SD).

## 3. Results and Discussion

### 3.1. Natural Aging of Samples

#### 3.1.1. Microscopic Images

**Figure 2:**
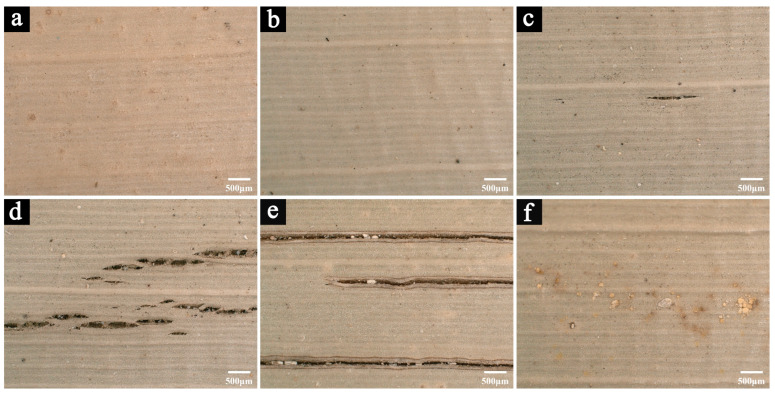
The microscopic images of the newly prepared samples (**a**) and samples naturally aged for 2 years (**b**), 5 years (**c**), 8 years (**d**), and 12 years (**e**,**f**).

Figure 2 shows the microscopic images of newly prepared samples and those stored in a cool and dry repository for 2, 5, 8, and 12 years, respectively.

After 2 years of natural storage (Figure 2b), the morphology of the samples shows no significant change compared with the newly prepared ones (Figure 2a), except for a slightly darker color. However, after 5 years of storage, fine cracks begin to appear on the sample surfaces (Figure 2c), and both the number and size of these cracks increase with prolonged storage time (Figure 2d,e).

Because the repository lacks stable temperature and humidity control, these cracks are most likely caused by fluctuations in the storage environment. Palm leaf manuscripts, being lignocellulosic materials, are highly sensitive to changes in temperature and humidity, which can induce physical deformation. Repeated expansion and contraction generate internal stress within the material, eventually leading to surface cracking [24]. As the storage time increases, such cracking becomes more pronounced and may further affect the mechanical and physical properties of the samples.

In addition, traces of mold growth can be observed in certain areas of the samples stored for 12 years (Figure 2f), confirming that short-term increases in relative humidity likely occurred in the repository, allowing temporary microbial proliferation.

#### 3.1.2. Mechanical Properties

**Figure 3:**
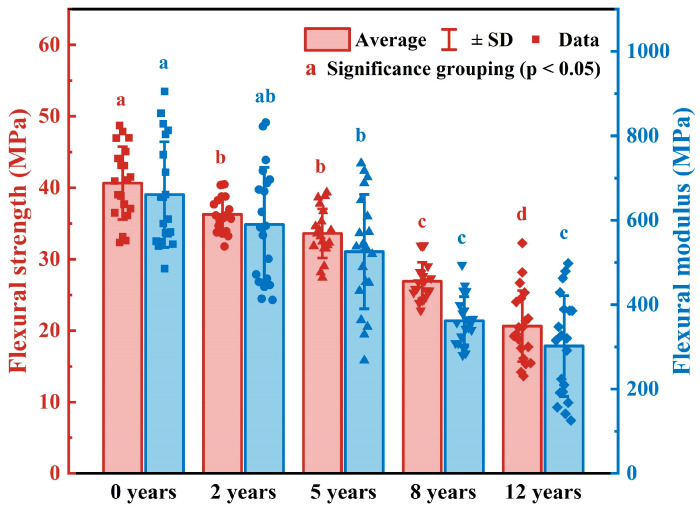
The flexural strength and flexural modulus of the naturally aged samples. Different letters indicate significant differences among groups based on Tukey’s HSD test (*p* < 0.05).

Figure 3 shows the flexural strength and flexural modulus of the newly prepared samples and those stored in a cool and dry repository for 2, 5, 8, and 12 years. Different letters indicate significant differences among groups based on Tukey’s HSD test (*p* < 0.05). Detailed pairwise comparison results are provided in Supplementary Table S1.

The results indicate that both the flexural strength and flexural modulus of the samples gradually decrease with increasing storage time. The flexural strength values of the newly prepared, 2-year, 5-year, 8-year, and 12-year naturally aged samples were 40.50 MPa, 36.28 MPa, 33.62 MPa, 26.90 MPa, and 20.89 MPa, respectively. Correspondingly, their flexural modulus values were 667.26 MPa, 605.10 MPa, 525.60 MPa, 362.11 MPa, and 332.09 MPa. Compared with the newly prepared samples, the flexural strength and flexural modulus of the samples stored for 12 years under natural conditions decreased by 48.42% and 50.23%, respectively.

This significant deterioration in mechanical properties is likely caused by the surface cracking observed in naturally aged samples. The degree of strength and modulus loss appears to correlate with the development of surface cracks in both length and area. These findings suggest that the cracking induced by physical deformation—resulting from fluctuations in temperature and humidity—leads to a progressive reduction in the mechanical performance of the material. With increasing storage time and continued crack propagation, the palm leaf manuscripts may become more brittle and prone to fracture or breakage under external forces.

The observed changes in mechanical properties during natural aging further highlight the critical importance of maintaining stable temperature and humidity conditions for the preservation of palm leaf manuscripts and other lignocellulosic cultural heritage materials.

#### 3.1.3. FT-IR

**Figure 4:**
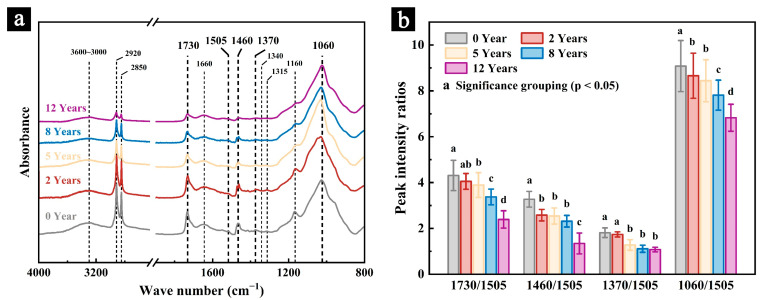
The infrared spectra (**a**) and the ratio of characteristic peak intensities (**b**) of the naturally aged samples. Different letters indicate significant differences among groups based on Tukey’s HSD test (*p* < 0.05).

Figure 4 shows the infrared spectra of the newly prepared samples and those stored in a cool and dry repository for 2, 5, 8, and 12 years, along with the variations in the relative intensities of the characteristic peaks of cellulose and hemicelluloses. Different letters indicate significant differences among groups based on Tukey’s HSD test (*p* < 0.05). Detailed pairwise comparison results are provided in Supplementary Table S2. The functional groups and their assignments corresponding to the characteristic peaks and bands in the infrared spectra of the samples are detailed in Table 1.

The results show that the overall absorption intensity of the infrared spectra gradually decreases with increasing storage time (Figure 4a). Meanwhile, the relative intensities of the characteristic peaks corresponding to cellulose and hemicelluloses also diminish progressively (Figure 4b). For the samples stored under natural conditions for 12 years, the peak intensity ratios of I_1730_/I_1505_, I_1460_/I_1505_, I_1370_/I_1505_, and I_1060_/I_1505_ decreased by 40.54%, 48.91%, 30.53%, and 24.82%, respectively, compared with the newly prepared samples. These results indicate that the main chemical components—cellulose and hemicelluloses—underwent degradation during natural aging. Such degradation is likely caused by hydrolysis reactions triggered by residual acidic substances from the boiling process used during sample preparation or by microbial erosion observed on the sample surfaces [28]. The higher degree of degradation observed in hemicelluloses suggests that it is less stable than cellulose and more susceptible to environmental factors in natural conditions.

Previous studies have shown that hemicellulose degradation contributes to the loss of mechanical properties in lignocellulosic materials [24], which is consistent with the mechanical test results of this study. Furthermore, these findings underscore the importance of maintaining stable temperature and humidity conditions for the preservation of palm leaf manuscripts and other cultural heritage materials composed of lignocellulosic fibers.

**Table 1:** Characteristic peaks in the infrared spectrum of the samples and corresponding functional group ascriptions.

Wave Number (cm^−1^)	Functional Groups Ascriptions
3600~3000	Stretching vibration by the hydroxyl on cellulose and the intermolecular hydrogen bonds [29]
3000~2800	Stretching vibration by the methyl and methylene groups on cellulose, hemicelluloses, and lignin [30,31]
1730	Stretching vibration by the carbonyl group on hemicelluloses [25,26]
1660	Stretching vibration by the carbonyl group on deconjugated carbonyl ketone in lignin [30,32]
1550~1500	Backbone vibration of butyl propane in butyl lignin [26,33]
1460	Stretching vibration by the methylene group on hemicelluloses [25,26]
1450	Bending vibration by the methylene group in lignin and the hydroxyl group in cellulose [34,35]
1370	Bending vibration by the methyl group on cellulose [26,27]
1330	Bending vibration by the methyl group on methoxy in amorphous cellulose [34,35]
1315	Bending vibration by the methylene group on crystalline cellulose [34,36]
1160	Bending vibration by the glycosidic bond on glucopyranose, carbohydrate, and crystalline cellulose [31,34]
1060	Bending vibration by the glycosidic bond on cellulose [26,27]

### 3.2. Simulated Aging of Samples

#### 3.2.1. Microscopic Images

**Figure 5:**
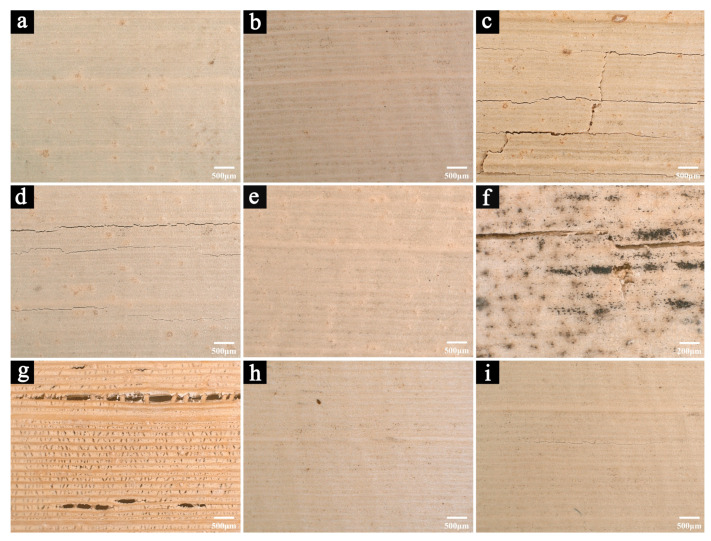
The microscopic images of the newly prepared samples after 200 days of simulated aging under different environmental conditions: −20 °C (**a**), 25 °C (**b**), 100 °C (**c**), 10% RH (**d**), 50% RH (**e**), 90% RH (**f**), UV (**g**), VIS (**h**), and IR (**i**).

Figure 5 shows the microscopic images of newly prepared samples after 200 days of aging under different temperature (−20 °C, 25 °C, and 100 °C), relative humidity (10% RH, 50% RH, and 90% RH), and light radiation (UV, VIS, and IR) conditions.

Samples aged at 100 °C exhibited severe surface cracking, with longitudinal and transverse cracks interwoven across the surface, and some crack edges curling up, posing a risk of flaking (Figure 5c). In addition, the color of the samples changed significantly compared with that before aging, turning distinctly yellow. It is generally accepted that high-temperature environments cause moisture loss and shrinkage in lignocellulosic materials. During the shrinkage process, differences in volumetric contraction along different directions generate internal stresses, which in turn lead to cracking. Previous studies have shown that the longitudinal fibers of palm leaf manuscripts possess higher mechanical strength than the transverse fibers, resulting in the formation of transverse cracks when deformation occurs [37]. The higher number and density of transverse cracks on the surface of the samples aged at 100 °C indicate that such cracking was mainly caused by deformation induced by shrinkage due to thermal dehydration. On the other hand, the significant color change in the samples may be attributed to oxidation of lignin under high-temperature conditions, leading to the formation of colored groups such as quinones and ketones [17,18].

Samples aged under 10% RH (Figure 5d) and under infrared radiation (Figure 5i) also exhibited transverse cracking, although less severe than that observed at 100 °C. These cracks were likely caused by deformation resulting from dehydration and shrinkage induced by dry conditions and the thermal effect of infrared radiation, respectively. This further confirms that transverse cracks tend to form first in palm leaf manuscripts when deformation occurs.

Samples aged under 90% RH developed extensive mold growth due to excessive humidity. Figure 5f shows a microscopic image of the sample surface after the removal of mold. Numerous spot-like traces of fungal growth remained on the surface, along with pores and cracks caused by microbial degradation. It is likely that hyphae penetrated through these structures into the interior of the sample, leading to further biodeterioration.

Samples aged under UV radiation exhibited the most severe cracking among all conditions. The cracks not only appeared on the surface but also extended through the entire sample, exposing the internal fiber structure (Figure 5g). The sample color also changed markedly compared with that before aging. This phenomenon occurs because UV radiation possesses high energy capable of directly breaking the chemical bonds in cellulose, hemicelluloses, and lignin molecules, leading to their degradation [38,39]. Prolonged exposure causes a gradual loss of these structural components, weakening the overall physical stability of the material and resulting in cracking. Furthermore, UV light promotes oxidation of lignin, causing methoxy and hydroxyl groups to react with oxygen to form chromophoric quinone and ketone structures, which lead to discoloration [38,39].

The cracking, curling, and microbial growth phenomena observed under different environmental aging conditions may cause varying degrees of deterioration in the mechanical properties of the samples. In contrast, samples aged at −20 °C (Figure 5a), 25 °C (Figure 5b), 50% RH (Figure 5e), and under visible light (Figure 5h) showed only slight color changes and no significant morphological alterations.

#### 3.2.2. Mechanical Properties

**Figure 6:**
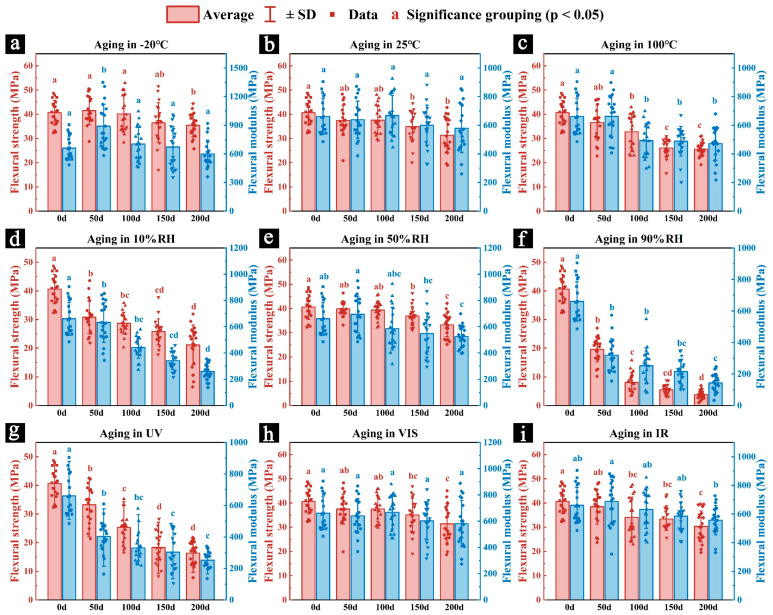
The changes in the flexural strength and flexural modulus of the newly prepared samples during 200 days of simulated aging under different environmental conditions: −20 °C (**a**), 25 °C (**b**), 100 °C (**c**), 10% RH (**d**), 50% RH (**e**), 90% RH (**f**), UV (**g**), VIS (**h**), and IR (**i**). Different letters indicate significant differences among groups based on Tukey’s HSD test (*p* < 0.05).

**Figure 7:**
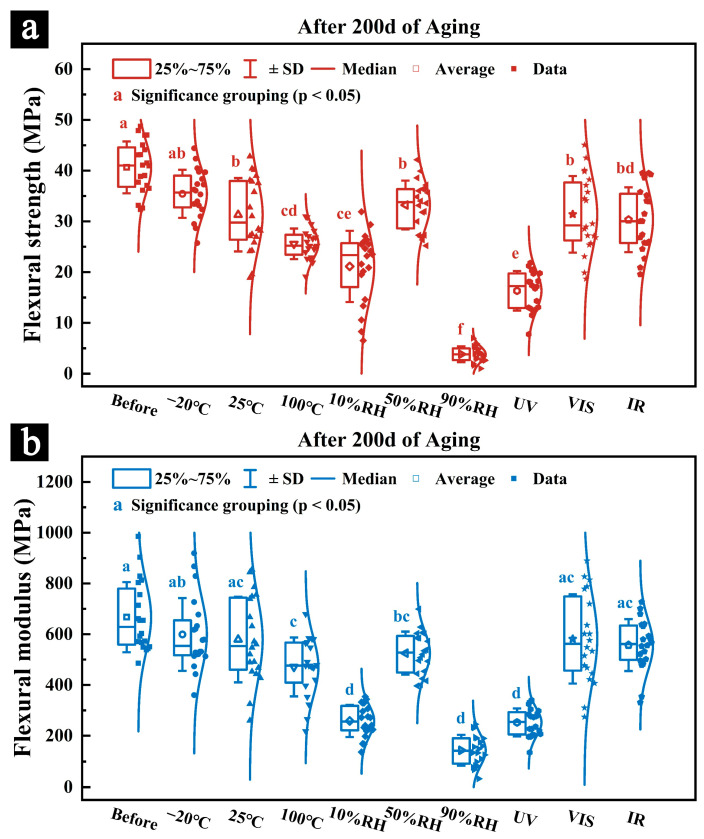
The flexural strength (**a**) and flexural modulus (**b**) of the newly prepared samples after 200 days of simulated aging under different environmental conditions. Different letters indicate significant differences among groups based on Tukey’s HSD test (*p* < 0.05).

Figure 6 shows the changes in flexural strength and flexural modulus of newly prepared samples during 200 days of aging under different conditions of temperature (−20 °C, 25 °C, and 100 °C), relative humidity (10% RH, 50% RH, and 90% RH), and light radiation (UV, VIS, and IR). Figure 7 shows the flexural strength and flexural modulus of the samples after 200 days of aging under these conditions. Different letters indicate significant differences among groups based on Tukey’s HSD test (*p* < 0.05). Detailed pairwise comparison results are provided in Supplementary Tables S3–S13.

The variations in flexural strength and flexural modulus of the samples aged under different temperature conditions showed distinct trends. As shown in Figure 6a, samples aged at −20 °C exhibited a temporary increase in both flexural strength and flexural modulus at the early stage of aging, reaching values slightly higher than those before aging, followed by a gradual decline as aging progressed. This unusual phenomenon may be attributed to the freezing of water within the samples, which helps maintain the structural stability of cellulose fibers, thereby enhancing the mechanical strength and stiffness in the initial stage. However, as aging continues, the growth and rupture of ice crystals may damage the cellulose fiber network, leading to a subsequent decrease in mechanical performance [40,41]. In contrast, the samples aged at 25 °C (Figure 6b) and 100 °C (Figure 6c) showed a continuous decrease in flexural strength and modulus over time, with the decline being more rapid and pronounced at 100 °C, likely due to severe surface cracking. After 200 days of aging, the flexural strengths of samples aged at −20 °C, 25 °C, and 100 °C were 35.42 MPa, 31.59 MPa, and 25.57 MPa, respectively (Figure 7a), corresponding to reductions of 12.54%, 22.00%, and 36.86% compared with unaged samples. The corresponding flexural modulus values were 598.92 MPa, 579.08 MPa, and 470.99 MPa (Figure 7b), representing decreases of 10.24%, 13.22%, and 29.41%, respectively. These results indicate that high-temperature environments not only cause deformation and cracking due to dehydration shrinkage but also significantly reduce the mechanical properties of the samples. Considering the potential damage from ice crystal expansion and rupture under low temperatures, storing palm leaf manuscripts at a moderate temperature (around 25 °C) may be more beneficial for preserving their mechanical integrity.

Regarding relative humidity, the flexural strength and modulus of samples aged under different RH conditions also showed varying degrees of reduction (Figure 6d–f). The samples aged at 90% RH experienced the most severe and rapid deterioration, likely due to microbial attack that caused both physical damage and chemical degradation [28]. Excessive moisture absorption in humid environments may also weaken the mechanical strength and stiffness. After 200 days, the flexural strengths of the samples aged at 10% RH, 50% RH, and 90% RH were 21.13 MPa, 33.22 MPa, and 3.82 MPa, respectively (Figure 7a), corresponding to reductions of 47.83%, 17.98%, and 90.57%. The flexural modulus values were 258.16 MPa, 535.31 MPa, and 143.81 MPa (Figure 7b), representing reductions of 61.31%, 19.78%, and 78.45%. These results suggest that excessively dry environments can cause shrinkage-induced deformation and cracking similar to those in high-temperature conditions, while overly humid environments promote microbial growth and chemical degradation of the main components. In contrast, samples aged at 50% RH maintained relatively high flexural strength and modulus, indicating that moderate humidity conditions are more favorable for preserving the mechanical properties of palm leaf manuscripts.

In terms of light radiation, samples aged under different illumination conditions also exhibited varying degrees of mechanical degradation (Figure 6g–i). The samples exposed to UV radiation showed the fastest and most pronounced decline in mechanical performance, primarily due to the photochemical degradation of cellulose and other major components, as well as structural damage (Figure 5g) [38,39]. After 200 days, the flexural strengths of samples aged under UV, visible, and infrared radiation were 16.32 MPa, 32.49 MPa, and 30.32 MPa, respectively (Figure 7a), representing reductions of 59.71%, 19.78%, and 25.14%. Their corresponding flexural modulus values were 252.29 MPa, 561.38 MPa, and 557.17 MPa (Figure 7b), corresponding to decreases of 62.19%, 15.92%, and 16.50%. These results indicate that UV radiation significantly reduces the mechanical properties of the samples, while visible and infrared light have comparatively minor effects.

Overall, the humid environment had the most significant impact on the mechanical performance of the samples. The samples aged at 90% RH showed dramatic decreases in flexural strength and modulus—90.57% and 78.45%, respectively—almost completely losing their mechanical integrity. Samples aged under UV radiation and in dry environments also exhibited substantial deterioration. Although low-temperature aging temporarily improved mechanical properties, prolonged exposure could still cause internal damage and mechanical degradation. In comparison, samples aged under moderate, non-extreme conditions (25 °C, 50% RH, and visible light) maintained the best mechanical performance.

#### 3.2.3. FT-IR

**Figure 8:**
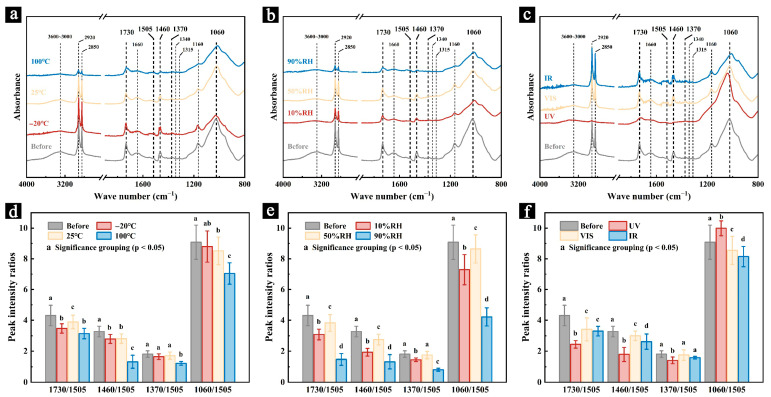
The infrared spectra and the ratio of characteristic peak intensities of the newly prepared samples after 200 days of simulated aging under different conditions of temperature (**a**,**d**), relative humidity (**b**,**e**), and light radiation (**c**,**f**). Different letters indicate significant differences among groups based on Tukey’s HSD test (*p* < 0.05).

Figure 8 shows the infrared spectra of newly prepared samples after 200 days of aging under different conditions of temperature (−20 °C, 25 °C, and 100 °C), relative humidity (10% RH, 50% RH, and 90% RH), and light radiation (UV, VIS, and IR), along with the variations in the relative intensities of the characteristic peaks of cellulose and hemicelluloses. Different letters indicate significant differences among groups based on Tukey’s HSD test (*p* < 0.05). Detailed pairwise comparison results are provided in Supplementary Tables S14 and S15.

The results show that after 200 days of aging, the overall absorption intensity of the infrared spectra of all samples decreased to varying degrees (Figure 8a–c), and a noticeable reduction in the relative intensities of the characteristic peaks of cellulose and hemicelluloses was observed in most samples (Figure 8d–f).

After 200 days of aging, the samples aged at 100 °C showed a significant decrease in the relative intensities of the characteristic peaks of cellulose and hemicelluloses (Figure 8d). The peak intensity ratios of I_1730_/I_1505_, I_1460_/I_1505_, I_1370_/I_1505_, and I_1060_/I_1505_ decreased by 27.16%, 59.63%, 25.83%, and 22.47%, respectively, compared to the unaged samples. These results indicate that under high-temperature conditions, the main chemical components of the samples (such as cellulose and hemicelluloses) underwent significant degradation. Moreover, the degradation of hemicelluloses was much more pronounced than that of cellulose, suggesting that hemicelluloses have lower thermal stability and are more prone to degradation. In contrast, the samples aged at low temperatures (−20 °C) and room temperature (25 °C) also exhibited some degradation, but the extent of degradation was lower than that observed under high-temperature conditions. After 200 days of aging, the peak intensity ratios of I_1730_/I_1505_, I_1460_/I_1505_, I_1370_/I_1505_, and I_1060_/I_1505_ for the samples aged at −20 °C decreased by 13.55%, 14.70%, 8.12%, and 3.12%, respectively, and for those aged at 25 °C, the reductions were 9.77%, 13.09%, 5.57%, and 6.39%. These results suggest that low and room temperature conditions had a lesser impact on the chemical structure of the samples compared to high-temperature environments.

In a dry environment (10% RH), noticeable degradation of the chemical components was also observed. After 200 days of aging, the peak intensity ratios of I_1730_/I_1505_, I_1460_/I_1505_, I_1370_/I_1505_, and I_1060_/I_1505_ decreased by 27.55%, 39.65%, 19.83%, and 16.71%, respectively (Figure 8e). This change is likely due to the rapid shrinkage of samples during moisture loss, which led to cracking (Figure 5d). These cracks make the cellulose and hemicelluloses more susceptible to environmental factors (such as temperature and oxygen), thus accelerating their degradation process [42]. In contrast, the samples aged at 90% RH exhibited a more significant decrease in peak intensity ratios, with reductions of 71.31%, 59.65%, 55.27%, and 52.88%, respectively, after 200 days of aging. This indicates that microbial activity, promoted by the high humidity, likely contributed to the degradation of the samples’ main chemical components. Microorganisms secrete specific enzymes that directly degrade cellulose and hemicelluloses or produce organic acids through their metabolism, which catalyze the hydrolysis of these materials [28]. In comparison, the samples aged at 50% RH retained relatively strong absorption peaks. After 200 days, the peak intensity ratios of I_1730_/I_1505_, I_1460_/I_1505_, I_1370_/I_1505_, and I_1060_/I_1505_ only decreased by 11.16%, 15.93%, 3.31%, and 4.85%, respectively, compared to the unaged samples.

Under UV radiation, the changes in the cellulose and hemicellulose content of the samples were also significant. The peak intensity ratios of I_1730_/I_1505_, I_1460_/I_1505_, and I_1370_/I_1505_ decreased by 43.40%, 45.18%, and 22.14%, respectively, while the ratio of I_1060_/I_1505_ increased by 10.01% (Figure 8f). These results suggest that under UV radiation, the lignin in the samples underwent significant degradation. Although cellulose and hemicelluloses also degraded under UV light, the extensive breakdown of lignin caused the relative intensity of the cellulose and hemicellulose peaks to decrease less drastically and even resulted in an increase in the relative intensity of some peaks [38,39]. In contrast, samples aged under visible light and infrared radiation retained relatively strong absorption intensities, indicating that the degradation of their main components was less significant. After 200 days of aging, the peak intensity ratios of I_1730_/I_1505_, I_1460_/I_1505_, I_1370_/I_1505_, and I_1060_/I_1505_ for the samples aged under visible light decreased by 18.87%, 8.65%, 3.09%, and 5.81%, while intensity ratios for the samples that aged under infrared radiation decreased by 23.52%, 19.63%, 12.58%, and 10.29%, respectively. The degradation of the main chemical components of the samples aged under infrared light may be due to the thermal effects of infrared radiation, though its impact was far less significant than that of high-temperature conditions.

Previous studies have shown that the degradation of major chemical components, particularly the loss of hemicelluloses, leads to a significant reduction in the mechanical properties of lignocellulosic materials, and consequently, the deterioration of the physical properties of palm leaf manuscripts. This is consistent with the mechanical performance results of the samples, where those aged under high temperature, dry conditions, high humidity, and UV radiation exhibited significant losses in mechanical properties due to the combined effects of chemical degradation and physical damage to their structures (Figure 6 and Figure 7).

### 3.3. Discussion and Prospect

The results show that under different environmental conditions, both the bending strength and bending modulus of the samples decrease to varying degrees during the aging process, and the degradation rate is closely related to the environmental conditions.

At low temperatures (−20 °C), samples displayed a temporary initial rise in flexural strength and modulus during early aging, owing to frozen internal moisture that stabilized the cellulose fiber network; however, prolonged exposure triggered ice crystal growth and rupture, damaging the fiber structure and reducing mechanical performance. By contrast, properties declined steadily at room temperature (25 °C) and rapidly at high temperature (100 °C), with the latter showing particularly severe degradation from dehydration-induced shrinkage, cracking, and thermal breakdown of cellulose and hemicelluloses.

Relative humidity exerted a notable influence. Samples aged in high-humidity environments (90% RH) experienced the most severe mechanical deterioration, mainly from microbial activity and moisture-driven hydrolysis that hastened cellulose and hemicellulose degradation. Extreme dryness (10% RH) likewise caused cracking and structural shrinkage, impairing mechanical integrity. By contrast, moderate humidity (50% RH) slowed chemical degradation and preserved flexural properties, proving the optimal range for preservation.

Light radiation also critically influenced degradation behavior. UV radiation triggered extensive photochemical degradation of lignin, cellulose, and hemicelluloses in samples, causing substantial losses in mechanical strength and stiffness. Visible and infrared light produced milder effects, with infrared primarily driven by thermal influences rather than direct photochemical reactions.

FT-IR analysis confirmed that cellulose and hemicellulose degradation were the main drivers of mechanical decline, with severe chemical changes under high temperature, extreme humidity, and UV radiation closely matching sharp drops in flexural strength and modulus, indicating a strong link between chemical composition and mechanical properties.

These findings indicate that relatively moderate and stable conditions (such as 25 °C, 50% RH, and limited light exposure) can more effectively preserve the structural integrity and mechanical properties of palm leaf manuscripts compared with environments that are excessively cold, hot, humid, or dry. Therefore, it is necessary to implement preventive conservation measures to maintain controlled environmental conditions for the preservation of these valuable cultural heritages—particularly in countries or regions that house large collections of palm leaf manuscripts, such as the humid and hot areas of India, Myanmar, and Yunnan in China, as well as the cold and dry regions of Tibet in China.

Although this study provides preliminary insights into the effects of temperature, humidity, and light radiation on the mechanical properties of palm leaf manuscripts, it is subject to certain limitations. Primarily, the research focused solely on the constant values of individual environmental factors, without considering the comprehensive impacts arising from the interactions of multiple factors, due to experimental constraints. This approach may not fully capture the complex real-world conditions encountered in the preservation of palm leaf manuscripts. Additionally, factors such as microorganisms and air pollutants should not be overlooked.

Future investigations should delve deeper into the coupled effects of various environmental factors on the mechanical properties and other physicochemical characteristics of palm leaf manuscripts, including the superposition of multiple factors and fluctuations in temperature and humidity. These efforts will offer more profound scientific foundations for the long-term and preventive conservation of palm leaf manuscripts.

## 4. Conclusions

This study systematically investigated the effects of natural aging and simulated aging under different temperature, humidity, and light radiation conditions on the mechanical properties of the palm leaf manuscripts. The results revealed that both flexural strength and flexural modulus of the samples decreased to varying degrees during aging, and the degradation rate strongly depended on the environmental conditions.

- Temperature: Low-temperature environments enhance the mechanical properties of palm leaf manuscripts in the short term, but the accompanying growth and rupture of ice crystals damage the fibers and reduce the mechanical properties of palm leaf manuscripts. In contrast, the mechanical properties of palm leaf manuscripts decline rapidly in high-temperature environments, primarily due to dehydration-induced shrinkage, cracking, and thermal decomposition of hemicellulose.
- Relative Humidity: Humid environments accelerate the degradation of cellulose and hemicellulose through microbial activity and moisture-driven hydrolysis, leading to the maximum deterioration of the mechanical properties of palm leaf manuscripts. Dry environments, meanwhile, impair their mechanical properties by inducing cracking and shrinkage in palm leaf manuscripts.
- Light Radiation: Ultraviolet irradiation triggers extensive photochemical degradation of lignin, cellulose, and hemicellulose in palm leaf manuscripts, resulting in significant losses in mechanical properties. Infrared light produces milder effects, primarily influencing the mechanical properties of palm leaf manuscripts through its thermal impact.
- FT-IR analysis confirmed a strong link between chemical composition and mechanical properties.

Overall, moderate and stable conditions (around 25 °C, 50% RH, and limited light exposure) best preserve the structural integrity and mechanical properties of palm leaf manuscripts. For long-term conservation, store them in controlled temperature and humidity environments, avoiding direct light exposure and fluctuations, to minimize degradation and extend their lifespan.

## Data Availability

The original contributions presented in this study are included in the article/Supplementary Material. Further inquiries can be directed to the corresponding author.

## Supplementary Materials

The following supporting information can be downloaded at https://www.mdpi.com/article/10.3390/polym17233229/s1. Tables S1–S16: The Tukey HSD pairwise comparisons among groups.

## References

[1] Kumar D.U., Sreekumar G., Athvankar U. (2009). Traditional writing system in southern India—Palm leaf manuscripts. Des. Thoughts.

[2] Panigrahi A.K., Litt D. (2018). Odia Script in Palm-Leaf Manuscripts. J. Humanit. Soc. Sci..

[3] Meher R. (2009). Tradition of palm leaf manuscripts in Orissa. Orissa Rev..

[4] Sharma D., Singh M., Krist G., Velayudhan N.M. (2018). Structural characterisation of 18th century Indian Palm leaf manuscripts of India. Int. J. Conserv. Sci..

[5] Sharma D., Singh M.R., Dighe B. (2018). Chromatographic Study on Traditional Natural Preservatives Used for Palm Leaf Manuscripts in India. Restaurator.

[6] Singh M.R., Sharma D. (2020). Investigation of Pigments on an Indian Palm Leaf Manuscript (18th–19th century) by SEM-EDX and other Techniques. Restaurator.

[7] Agrawal O.P. (1984). Conservation of Manuscripts and Paintings of South-East Asia.

[8] Sah A. (2002). Palm Leaf Manuscripts of the World: Material, Technology and Conservation. Stud. Conserv..

[9] Alexander T.J., Kumar S.S. (2020). A novel binarization technique based on Whale Optimization Algorithm for better restoration of palm leaf manuscript. J. Ambient Intell. Humaniz. Comput..

[10] Subramani K., Subramaniam M. (2021). Creation of original Tamil character dataset through segregation of ancient palm leaf man-uscripts in medicine. Expert Syst..

[11] Sabeenian R., Paramasivam M., Anand R., Dinesh P. (2019). Palm-leaf manuscript character recognition and classification using convolutional neural networks. Computing and Network Sustainability: Proceedings of IRSCNS 2018.

[12] Zhang M., Song X., Wang J., Lyu X. (2022). Preservation characteristics and restoration core technology of palm leaf manuscripts in Potala Palace. Arch. Sci..

[13] Zhang W., Wang S., Han L., Guo H. (2025). Study on the hygroscopicity and kinetic and thermodynamic properties of ancient Tibetan Palm Leaf Manuscripts. npj Herit. Sci..

[14] Wiland J., Brown R., Fuller L., Havelock L., Johnson J., Kenn D., Kralka P., Muzart M., Pollard J., Snowdon J. (2022). A literature review of palm leaf manuscript conservation—Part 1: A historic overview, leaf preparation, materials and media, palm leaf manuscripts at the British Library and the common types of damage. J. Inst. Conserv..

[15] Wiland J., Brown R., Fuller L., Havelock L., Johnson J., Kenn D., Kralka P., Muzart M., Pollard J., Snowdon J. (2023). A literature review of palm leaf manuscript conservation—Part 2: Historic and current conservation treatments, boxing and storage, reli-gious and ethical issues, recommendations for best practice. J. Inst. Conserv..

[16] Yi X., Zhang M., Huang Y., Wang X., Zhang Y., Lv S. (2024). Study on the material properties and deterioration mechanism of palm leaves. Restaurator.

[17] Graminski E.L., Parks E.J., Toth E.E. (1979). The Effects of Temperature and Moisture on the Accelerated Aging of Paper. Restaurator.

[18] Liu X.Y., Timar M.C., Varodi A.M., Sawyer G. (2017). An Investigation of Accelerated Temperature-Induced Ageing of Four Wood Species: Colour and FTIR. Wood Sci. Technol..

[19] Bylund Melin C., Hagentoft C.-E., Holl K., Nik V.M., Kilian R. (2018). Simulations of Moisture Gradients in Wood Subjected to Changes in Relative Humidity and Temperature Due to Climate Change. Geosciences.

[20] Kim M.-J., Choi Y.-S., Oh J.-J., Kim G.-H. (2020). Experimental investigation of the humidity effect on wood discoloration by selected mold and stain fungi for a proper conservation of wooden cultural heritages. J. Wood Sci..

[21] Botti S., Di Lazzaro P., Flora F., Mezi L., Murra D. (2024). Raman spectral mapping reveal molecular changes in cellulose aging induced by ultraviolet and extreme ultraviolet radiation. Cellulose.

[22] Mitsui K., Takada H., Sugiyama M., Hasegawa R. (2001). Changes in the Properties of Light-Irradiated Wood with Heat Treatment: Part 1. Effect of Treatment Conditions on the Change in Color. Holzforschung.

[23] Zhang W., Wang S., Han L., Guo H. (2025). Aging effects of relative humidity on palm leaf manuscripts and optimal humidity conditions for preservation. npj Herit. Sci..

[24] Zhang W., Wang S., Guo H. (2024). Influence of Relative Humidity on the Mechanical Properties of Palm Leaf Manuscripts: Short-Term Effects and Long-Term Aging. Molecules.

[25] Schwanninger M., Rodrigues J.C., Pereira H., Hinterstoisser B. (2004). Effects of short-time vibratory ball milling on the shape of FT-IR spectra of wood and cellulose. Vib. Spectrosc..

[26] Marchessault R.H. (1962). Application of infra-red spectroscopy to cellulose and wood polysaccharides. Pure Appl. Chem..

[27] Pandey K.K., Pitman A.J. (2003). FTIR studies of the changes in wood chemistry following decay by brown-rot and white-rot fungi. Int. Biodeterior. Biodegrad..

[28] Chu S., Lin L., Tian X. (2024). Analysis of Aspergillus niger isolated from ancient palm leaf manuscripts and its deterioration mechanisms. npj Herit. Sci..

[29] Merino D., Athanassiou A. (2023). Alkaline hydrolysis of biomass as an alternative green method for bioplastics preparation: In situ cellulose nanofibrillation. J. Chem. Eng..

[30] Boukir A., Guiliano M., Asia L., El Hallaoui A., Mille G. (1998). A fraction to fraction study of photo-oxidation of BAL 150 crude oil asphaltenes. Analusis.

[31] Biswas S., Rahaman T., Gupta P., Mitra R., Dutta S., Kharlyngdoh E., Guha S., Ganguly J., Pal A., Das M. (2022). Cellulose and lignin profiling in seven, economically important bamboo species of India by anatomical, biochemical, FTIR spectroscopy and thermogravimetric analysis. Biomass Bioenergy.

[32] Kotov N., Larsson P.A., Jain K., Abitbol T., Cernescu A., Wågberg L., Johnson C.M. (2023). Elucidating the fine-scale structural morphology of nanocellulose by nano infrared spectroscopy. Carbohydr. Polym..

[33] Kumar P., Miller K., Kermanshahi-Pour A., Brar S.K., Beims R.F., Xu C.C. (2022). Nanocrystalline cellulose derived from spruce wood: Influence of process parameters. Int. J. Biol. Macromol..

[34] Bouramdane Y., Haddad M., Mazar A., Aît Lyazidi S., Oudghiri Hassani H., Boukir A. (2024). Aged Lignocellulose Fibers of Cedar Wood (9th and 12th Century): Structural Investigation Using FTIR-Deconvolution Spectroscopy, X-Ray Diffraction (XRD), Crystallinity Indices, and Morphological SEM Analyses. Polymers.

[35] Broda M., Popescu C.-M. (2019). Natural decay of archaeological oak wood versus artificial degradation processes—An FT-IR spectroscopy and X-ray diffraction study. Spectrochim. Acta A Mol. Biomol. Spectrosc..

[36] Boukir A., Fellak S., Doumenq P. (2019). Structural characterization of Argania spinosa Moroccan wooden artifacts during natural degradation progress using infrared spectroscopy (ATR-FTIR) and X-Ray diffraction (XRD). Heliyon.

[37] Chu S., Lin L., Tian X. (2023). Evaluation of the Deterioration State of Historical Palm Leaf Manuscripts from Burma. Forests.

[38] Zhang X., Yan Y., Yao J., Jin S., Tang Y. (2023). Chemistry directs the conservation of paper cultural relics. Polymer Degrad. Stab..

[39] Jablonsky M., Šima J. (2021). Oxidative degradation of paper—A minireview. J. Cult. Herit..

[40] Wu J., Wang X., Fei B., Chen H. (2021). The mechanical properties and thermal conductivity of bamboo with freeze–thaw treatment. J. Wood Sci..

[41] Özkan O.E. (2022). Effect of freezing temperature on impact bending strength and shore-D hardness of some wood species. BioResources.

[42] Lv C., Liu J. (2023). Alkaline Degradation of Plant Fiber Reinforcements in Geopolymer: A Review. Molecules.

