# Supplementary material for: Effects of Environmental Factors on the Mechanical Properties of Palm Leaf Manuscripts: Natural Aging, Temperature, Relative Humidity, and Light Radiation
Source: Polymers (Basel). 2025 Dec 4;17(23):3229. doi: 10.3390/polym17233229 (PMC12693837; doi:10.3390/polym17233229)
Supplement: Supplementary file 1 [file polymers-17-03229-s001.zip › polymers-3973297-supplementary.pdf]

Supplementary Table

Table S1. The Tukey HSD pairwise comparisons among groups for the flexural strength and flexural modulus of samples aged at naturally aged.

Samples		Number	Comparison	p adj	Significance
0 years	flexural strength	0	1 vs 0	p < 0.01	**
2 years	flexural strength	1	2 vs 0	p < 0.001	***
5 years	flexural strength	2	2 vs 1	p > 0.05	n.s.
8 years	flexural strength	3	3 vs 0	p < 0.001	***
12 years	flexural strength	4	3 vs 1	p < 0.001	***
/		/	3 vs 2	p < 0.001	***
/		/	4 vs 0	p < 0.001	***
/		/	4 vs 1	p < 0.001	***
/		/	4 vs 2	p < 0.001	***
/		/	4 vs 3	p < 0.001	***
0 years	flexural modulus	0	1 vs 0	p > 0.05	n.s.
2 years	flexural modulus	1	2 vs 0	p < 0.01	**
5 years	flexural modulus	2	2 vs 1	p > 0.05	n.s.
8 years	flexural modulus	3	3 vs 0	p < 0.001	***
12 years	flexural modulus	4	3 vs 1	p < 0.001	***
/		/	3 vs 2	p < 0.001	***
/		/	4 vs 0	p < 0.001	***
/		/	4 vs 1	p < 0.001	***
/		/	4 vs 2	p < 0.001	***
/		/	4 vs 3	p > 0.05	n.s.

In the table, different numbers of asterisks denote levels of statistical significance: * indicates $p < 0.05$, ** indicates $p < 0.01$, *** indicates $p < 0.001$, and n.s. indicates $p \geq 0.05$, meaning the result is not statistically significant.

Table S2. The Tukey HSD pairwise comparisons among groups for the ratio of characteristic peak intensities of samples aged at naturally aged.

Samples		Number	Comparison	p adj	Significance
0 years	I_{1730}/I_{1505}	0	1 vs 0	$p > 0.05$	n.s.
2 years	I_{1730}/I_{1505}	1	2 vs 0	$p < 0.01$	**
5 years	I_{1730}/I_{1505}	2	2 vs 1	$p > 0.05$	n.s.
8 years	I_{1730}/I_{1505}	3	3 vs 0	$p < 0.001$	***
12 years	I_{1730}/I_{1505}	4	3 vs 1	$p < 0.001$	***
/		/	3 vs 2	$p < 0.001$	***
/		/	4 vs 0	$p < 0.001$	***
/		/	4 vs 1	$p < 0.001$	***
/		/	4 vs 2	$p < 0.001$	***
/		/	4 vs 3	$p < 0.001$	***
0 years	I_{1460}/I_{1505}	0	1 vs 0	$p < 0.001$	***
2 years	I_{1460}/I_{1505}	1	2 vs 0	$p < 0.001$	***
5 years	I_{1460}/I_{1505}	2	2 vs 1	$p > 0.05$	n.s.
8 years	I_{1460}/I_{1505}	3	3 vs 0	$p < 0.001$	***
12 years	I_{1460}/I_{1505}	4	3 vs 1	$p > 0.05$	n.s.
/		/	3 vs 2	$p > 0.05$	n.s.
/		/	4 vs 0	$p < 0.001$	***
/		/	4 vs 1	$p < 0.001$	***
/		/	4 vs 2	$p < 0.001$	***
/		/	4 vs 3	$p < 0.001$	***
0 years	I_{1370}/I_{1505}	0	1 vs 0	$p > 0.05$	n.s.
2 years	I_{1370}/I_{1505}	1	2 vs 0	$p < 0.001$	***
5 years	I_{1370}/I_{1505}	2	2 vs 1	$p < 0.001$	***
8 years	I_{1370}/I_{1505}	3	3 vs 0	$p < 0.001$	***
12 years	I_{1370}/I_{1505}	4	3 vs 1	$p < 0.001$	***
/		/	3 vs 2	$p > 0.05$	n.s.
/		/	4 vs 0	$p < 0.001$	***
/		/	4 vs 1	$p < 0.001$	***
/		/	4 vs 2	$p > 0.05$	n.s.
/		/	4 vs 3	$p > 0.05$	n.s.

0 years	I_{1060}/I_{1505}	0	1 vs 0	$p < 0.001$	***
2 years	I_{1060}/I_{1505}	1	2 vs 0	$p < 0.001$	***
5 years	I_{1060}/I_{1505}	2	2 vs 1	$p > 0.05$	n.s.
8 years	I_{1060}/I_{1505}	3	3 vs 0	$p < 0.001$	***
12 years	I_{1060}/I_{1505}	4	3 vs 1	$p < 0.001$	***
/		/	3 vs 2	$p < 0.001$	***
/		/	4 vs 0	$p < 0.001$	***
/		/	4 vs 1	$p < 0.001$	***
/		/	4 vs 2	$p < 0.001$	***
/		/	4 vs 3	$p < 0.001$	***

In the table, different numbers of asterisks denote levels of statistical significance: * indicates $p < 0.05$, ** indicates $p < 0.01$, *** indicates $p < 0.001$, and n.s. indicates $p \geq 0.05$, meaning the result is not statistically significant.

Table S3. The Tukey HSD pairwise comparisons among groups for the flexural strength and flexural modulus of samples aged at -20 ℃.

Samples		Number	Comparison	p adj	Significance
0 days	flexural strength	0	1 vs 0	p > 0.05	n.s.
50 days	flexural strength	1	2 vs 0	p > 0.05	n.s.
100 days	flexural strength	2	2 vs 1	p > 0.05	n.s.
150 days	flexural strength	3	3 vs 0	p > 0.05	n.s.
200 days	flexural strength	4	3 vs 1	p > 0.05	n.s.
/		/	3 vs 2	p > 0.05	n.s.
/		/	4 vs 0	p < 0.05	*
/		/	4 vs 1	p < 0.05	*
/		/	4 vs 2	p < 0.05	*
/		/	4 vs 3	p > 0.05	n.s.
0 days	flexural modulus	0	1 vs 0	p < 0.001	***
50 days	flexural modulus	1	2 vs 0	p > 0.05	n.s.
100 days	flexural modulus	2	2 vs 1	p < 0.001	***
150 days	flexural modulus	3	3 vs 0	p > 0.05	n.s.
200 days	flexural modulus	4	3 vs 1	p < 0.001	***
/		/	3 vs 2	p > 0.05	n.s.
/		/	4 vs 0	p > 0.05	n.s.
/		/	4 vs 1	p < 0.001	***
/		/	4 vs 2	p > 0.05	n.s.
/		/	4 vs 3	p > 0.05	n.s.

In the table, different numbers of asterisks denote levels of statistical significance: * indicates $p < 0.05$, ** indicates $p < 0.01$, *** indicates $p < 0.001$, and n.s. indicates $p \geq 0.05$, meaning the result is not statistically significant.

Table S4. The Tukey HSD pairwise comparisons among groups for the flexural strength and flexural modulus of samples aged at 25 ℃.

Samples		Number	Comparison	p adj	Significance
0 days	flexural strength	0	1 vs 0	$p > 0.05$	n.s.
50 days	flexural strength	1	2 vs 0	$p > 0.05$	n.s.
100 days	flexural strength	2	2 vs 1	$p > 0.05$	n.s.
150 days	flexural strength	3	3 vs 0	$p < 0.05$	*
200 days	flexural strength	4	3 vs 1	$p > 0.05$	n.s.
/		/	3 vs 2	$p > 0.05$	n.s.
/		/	4 vs 0	$p < 0.001$	***
/		/	4 vs 1	$p < 0.05$	*
/		/	4 vs 2	$p < 0.05$	*
/		/	4 vs 3	$p > 0.05$	n.s.
0 days	flexural modulus	0	1 vs 0	$p > 0.05$	n.s.
50 days	flexural modulus	1	2 vs 0	$p > 0.05$	n.s.
100 days	flexural modulus	2	2 vs 1	$p > 0.05$	n.s.
150 days	flexural modulus	3	3 vs 0	$p > 0.05$	n.s.
200 days	flexural modulus	4	3 vs 1	$p > 0.05$	n.s.
/		/	3 vs 2	$p > 0.05$	n.s.
/		/	4 vs 0	$p > 0.05$	n.s.
/		/	4 vs 1	$p > 0.05$	n.s.
/		/	4 vs 2	$p > 0.05$	n.s.
/		/	4 vs 3	$p > 0.05$	n.s.

In the table, different numbers of asterisks denote levels of statistical significance: * indicates $p < 0.05$, ** indicates $p < 0.01$, *** indicates $p < 0.001$, and n.s. indicates $p \geq 0.05$, meaning the result is not statistically significant.

Table S5. The Tukey HSD pairwise comparisons among groups for the flexural strength and flexural modulus of samples aged at 100 ℃.

Samples		Number	Comparison	p adj	Significance
0 days	flexural strength	0	1 vs 0	p > 0.05	n.s.
50 days	flexural strength	1	2 vs 0	p < 0.001	***
100 days	flexural strength	2	2 vs 1	p > 0.05	n.s.
150 days	flexural strength	3	3 vs 0	p < 0.001	***
200 days	flexural strength	4	3 vs 1	p < 0.001	***
/		/	3 vs 2	p < 0.01	**
/		/	4 vs 0	p < 0.001	***
/		/	4 vs 1	p < 0.001	***
/		/	4 vs 2	p < 0.001	***
/		/	4 vs 3	p > 0.05	n.s.
0 days	flexural modulus	0	1 vs 0	p > 0.05	n.s.
50 days	flexural modulus	1	2 vs 0	p < 0.001	***
100 days	flexural modulus	2	2 vs 1	p < 0.001	***
150 days	flexural modulus	3	3 vs 0	p < 0.001	***
200 days	flexural modulus	4	3 vs 1	p < 0.001	***
/		/	3 vs 2	p > 0.05	n.s.
/		/	4 vs 0	p < 0.001	***
/		/	4 vs 1	p < 0.001	***
/		/	4 vs 2	p > 0.05	n.s.
/		/	4 vs 3	p > 0.05	n.s.

In the table, different numbers of asterisks denote levels of statistical significance: * indicates $p < 0.05$, ** indicates $p < 0.01$, *** indicates $p < 0.001$, and n.s. indicates $p \geq 0.05$, meaning the result is not statistically significant.

Table S6. The Tukey HSD pairwise comparisons among groups for the flexural strength and flexural modulus of samples aged at 10%RH.

Samples		Number	Comparison	p adj	Significance
0 days	flexural strength	0	1 vs 0	$p < 0.001$	***
50 days	flexural strength	1	2 vs 0	$p < 0.001$	***
100 days	flexural strength	2	2 vs 1	$p > 0.05$	n.s.
150 days	flexural strength	3	3 vs 0	$p < 0.001$	***
200 days	flexural strength	4	3 vs 1	$p = 0.030$	*
/		/	3 vs 2	$p > 0.05$	n.s.
/		/	4 vs 0	$p < 0.001$	***
/		/	4 vs 1	$p < 0.001$	***
/		/	4 vs 2	$p < 0.001$	***
/		/	4 vs 3	$p > 0.05$	n.s.
0 days	flexural modulus	0	1 vs 0	$p < 0.001$	***
50 days	flexural modulus	1	2 vs 0	$p < 0.001$	***
100 days	flexural modulus	2	2 vs 1	$p > 0.05$	n.s.
150 days	flexural modulus	3	3 vs 0	$p < 0.001$	***
200 days	flexural modulus	4	3 vs 1	$p < 0.05$	*
/		/	3 vs 2	$p > 0.05$	n.s.
/		/	4 vs 0	$p < 0.001$	***
/		/	4 vs 1	$p < 0.001$	***
/		/	4 vs 2	$p < 0.001$	***
/		/	4 vs 3	$p > 0.05$	n.s.

In the table, different numbers of asterisks denote levels of statistical significance: * indicates $p < 0.05$, ** indicates $p < 0.01$, *** indicates $p < 0.001$, and n.s. indicates $p \geq 0.05$, meaning the result is not statistically significant.

Table S7. The Tukey HSD pairwise comparisons among groups for the flexural strength and flexural modulus of samples aged at 50%RH.

Samples		Number	Comparison	p adj	Significance
0 days	flexural strength	0	1 vs 0	$p > 0.05$	n.s.
50 days	flexural strength	1	2 vs 0	$p > 0.05$	n.s.
100 days	flexural strength	2	2 vs 1	$p > 0.05$	n.s.
150 days	flexural strength	3	3 vs 0	$p = 0.045$	*
200 days	flexural strength	4	3 vs 1	$p > 0.05$	n.s.
/		/	3 vs 2	$p > 0.05$	n.s.
/		/	4 vs 0	$p < 0.001$	***
/		/	4 vs 1	$p < 0.001$	***
/		/	4 vs 2	$p < 0.001$	***
/		/	4 vs 3	$p = 0.049$	*
0 days	flexural modulus	0	1 vs 0	$p > 0.05$	n.s.
50 days	flexural modulus	1	2 vs 0	$p > 0.05$	n.s.
100 days	flexural modulus	2	2 vs 1	$p > 0.05$	n.s.
150 days	flexural modulus	3	3 vs 0	$p > 0.05$	n.s.
200 days	flexural modulus	4	3 vs 1	$p < 0.01$	**
/		/	3 vs 2	$p > 0.05$	n.s.
/		/	4 vs 0	$p = 0.019$	*
/		/	4 vs 1	$p < 0.01$	**
/		/	4 vs 2	$p > 0.05$	n.s.
/		/	4 vs 3	$p > 0.05$	n.s.

In the table, different numbers of asterisks denote levels of statistical significance: * indicates $p < 0.05$, ** indicates $p < 0.01$, *** indicates $p < 0.001$, and n.s. indicates $p \geq 0.05$, meaning the result is not statistically significant.

Table S8. The Tukey HSD pairwise comparisons among groups for the flexural strength and flexural modulus of samples aged at 90% RH.

Samples		Number	Comparison	p adj	Significance
0 days	flexural strength	0	1 vs 0	$p < 0.001$	***
50 days	flexural strength	1	2 vs 0	$p < 0.001$	***
100 days	flexural strength	2	2 vs 1	$p < 0.001$	***
150 days	flexural strength	3	3 vs 0	$p < 0.001$	***
200 days	flexural strength	4	3 vs 1	$p < 0.001$	***
/		/	3 vs 2	$p > 0.05$	n.s.
/		/	4 vs 0	$p < 0.001$	***
/		/	4 vs 1	$p < 0.001$	***
/		/	4 vs 2	$p < 0.01$	**
/		/	4 vs 3	$p > 0.05$	n.s.
0 days	flexural modulus	0	1 vs 0	$p < 0.001$	***
50 days	flexural modulus	1	2 vs 0	$p < 0.001$	***
100 days	flexural modulus	2	2 vs 1	$p > 0.05$	n.s.
150 days	flexural modulus	3	3 vs 0	$p < 0.001$	***
200 days	flexural modulus	4	3 vs 1	$p = 0.010$	**
/		/	3 vs 2	$p > 0.05$	n.s.
/		/	4 vs 0	$p < 0.001$	***
/		/	4 vs 1	$p < 0.001$	***
/		/	4 vs 2	$p < 0.01$	**
/		/	4 vs 3	$p > 0.05$	n.s.

In the table, different numbers of asterisks denote levels of statistical significance: * indicates $p < 0.05$, ** indicates $p < 0.01$, *** indicates $p < 0.001$, and n.s. indicates $p \geq 0.05$, meaning the result is not statistically significant.

Table S9. The Tukey HSD pairwise comparisons among groups for the flexural strength and flexural modulus of samples aged at UV.

Samples		Number	Comparison	p adj	Significance
0 days	flexural strength	0	1 vs 0	$p < 0.001$	***
50 days	flexural strength	1	2 vs 0	$p < 0.001$	***
100 days	flexural strength	2	2 vs 1	$p < 0.001$	***
150 days	flexural strength	3	3 vs 0	$p < 0.001$	***
200 days	flexural strength	4	3 vs 1	$p < 0.001$	***
/		/	3 vs 2	$p < 0.001$	***
/		/	4 vs 0	$p < 0.001$	***
/		/	4 vs 1	$p < 0.001$	***
/		/	4 vs 2	$p < 0.001$	***
/		/	4 vs 3	$p > 0.05$	n.s.
0 days	flexural modulus	0	1 vs 0	$p < 0.001$	***
50 days	flexural modulus	1	2 vs 0	$p < 0.001$	***
100 days	flexural modulus	2	2 vs 1	$p > 0.05$	n.s.
150 days	flexural modulus	3	3 vs 0	$p < 0.001$	***
200 days	flexural modulus	4	3 vs 1	$p = 0.018$	*
/		/	3 vs 2	$p > 0.05$	n.s.
/		/	4 vs 0	$p < 0.001$	***
/		/	4 vs 1	$p < 0.001$	***
/		/	4 vs 2	$p > 0.05$	n.s.
/		/	4 vs 3	$p > 0.05$	n.s.

In the table, different numbers of asterisks denote levels of statistical significance: * indicates $p < 0.05$, ** indicates $p < 0.01$, *** indicates $p < 0.001$, and n.s. indicates $p \geq 0.05$, meaning the result is not statistically significant.

Table S10. The Tukey HSD pairwise comparisons among groups for the flexural strength and flexural modulus of samples aged at VIS.

Samples		Number	Comparison	p adj	Significance
0 days	flexural strength	0	1 vs 0	$p > 0.05$	n.s.
50 days	flexural strength	1	2 vs 0	$p > 0.05$	n.s.
100 days	flexural strength	2	2 vs 1	$p > 0.05$	n.s.
150 days	flexural strength	3	3 vs 0	$p = 0.047$	*
200 days	flexural strength	4	3 vs 1	$p > 0.05$	n.s.
/		/	3 vs 2	$p > 0.05$	n.s.
/		/	4 vs 0	$p < 0.001$	***
/		/	4 vs 1	$p = 0.023$	*
/		/	4 vs 2	$p = 0.022$	*
/		/	4 vs 3	$p > 0.05$	n.s.
0 days	flexural modulus	0	1 vs 0	$p > 0.05$	n.s.
50 days	flexural modulus	1	2 vs 0	$p > 0.05$	n.s.
100 days	flexural modulus	2	2 vs 1	$p > 0.05$	n.s.
150 days	flexural modulus	3	3 vs 0	$p > 0.05$	n.s.
200 days	flexural modulus	4	3 vs 1	$p > 0.05$	n.s.
/		/	3 vs 2	$p > 0.05$	n.s.
/		/	4 vs 0	$p > 0.05$	n.s.
/		/	4 vs 1	$p > 0.05$	n.s.
/		/	4 vs 2	$p > 0.05$	n.s.
/		/	4 vs 3	$p > 0.05$	n.s.

In the table, different numbers of asterisks denote levels of statistical significance: * indicates $p < 0.05$, ** indicates $p < 0.01$, *** indicates $p < 0.001$, and n.s. indicates $p \geq 0.05$, meaning the result is not statistically significant.

Table S11. The Tukey HSD pairwise comparisons among groups for the flexural strength and flexural modulus of samples aged at IR.

Samples		Number	Comparison	p adj	Significance
0 days	flexural strength	0	1 vs 0	$p > 0.05$	n.s.
50 days	flexural strength	1	2 vs 0	$p = 0.016$	*
100 days	flexural strength	2	2 vs 1	$p > 0.05$	n.s.
150 days	flexural strength	3	3 vs 0	$p < 0.01$	**
200 days	flexural strength	4	3 vs 1	$p > 0.05$	n.s.
/		/	3 vs 2	$p > 0.05$	n.s.
/		/	4 vs 0	$p < 0.001$	***
/		/	4 vs 1	$p < 0.01$	**
/		/	4 vs 2	$p = 0.022$	n.s.
/		/	4 vs 3	$p > 0.05$	n.s.
0 days	flexural modulus	0	1 vs 0	$p > 0.05$	n.s.
50 days	flexural modulus	1	2 vs 0	$p > 0.05$	n.s.
100 days	flexural modulus	2	2 vs 1	$p > 0.05$	n.s.
150 days	flexural modulus	3	3 vs 0	$p > 0.05$	n.s.
200 days	flexural modulus	4	3 vs 1	$p > 0.05$	n.s.
/		/	3 vs 2	$p > 0.05$	n.s.
/		/	4 vs 0	$p > 0.05$	n.s.
/		/	4 vs 1	$p < 0.05$	*
/		/	4 vs 2	$p > 0.05$	n.s.
/		/	4 vs 3	$p > 0.05$	n.s.

In the table, different numbers of asterisks denote levels of statistical significance: * indicates $p < 0.05$, ** indicates $p < 0.01$, *** indicates $p < 0.001$, and n.s. indicates $p \geq 0.05$, meaning the result is not statistically significant.

Table S12. The Tukey HSD pairwise comparisons among groups for the flexural strength of samples after 200 days under different environmental conditions.

Samples	Number	Comparison	p adj	Significance
Before	0	1 vs 0	$p > 0.05$	n.s.
-20℃	1	2 vs 0	$p < 0.001$	***
25℃	2	2 vs 1	$p > 0.05$	n.s.
100℃	3	3 vs 0	$p < 0.001$	***
10%RH	4	3 vs 1	$p < 0.001$	***
50%RH	5	3 vs 2	$p = 0.035$	*
90%RH	6	4 vs 0	$p < 0.001$	***
UV	7	4 vs 1	$p < 0.001$	***
VIS	8	4 vs 2	$p < 0.001$	***
IR	9	4 vs 3	$p > 0.05$	n.s.
/	/	5 vs 0	$p < 0.01$	**
/	/	5 vs 1	$p > 0.05$	n.s.
/	/	5 vs 2	$p > 0.05$	n.s.
/	/	5 vs 3	$p < 0.001$	***
/	/	5 vs 4	$p < 0.001$	***
/	/	6 vs 0	$p < 0.001$	***
/	/	6 vs 1	$p < 0.001$	***
/	/	6 vs 2	$p < 0.001$	***
/	/	6 vs 3	$p < 0.001$	***
/	/	6 vs 4	$p < 0.001$	***
/	/	6 vs 5	$p < 0.001$	***
/	/	7 vs 0	$p < 0.001$	***
/	/	7 vs 1	$p < 0.001$	***
/	/	7 vs 2	$p < 0.001$	***
/	/	7 vs 3	$p < 0.001$	***
		7 vs 4	$p > 0.05$	n.s.
		7 vs 5	$p < 0.001$	***
		7 vs 6	$p < 0.001$	***
		8 vs 0	$p < 0.001$	***
		8 vs 1	$p > 0.05$	n.s.

	8 vs 2	$p > 0.05$	n.s.
	8 vs 3	$p = 0.030$	*
	8 vs 4	$p < 0.001$	***
	8 vs 5	$p > 0.05$	n.s.
	8 vs 6	$p < 0.001$	***
	8 vs 7	$p < 0.001$	***
	9 vs 0	$p < 0.001$	***
	9 vs 1	$p > 0.05$	n.s.
	9 vs 2	$p > 0.05$	n.s.
	9 vs 3	$p > 0.05$	n.s.
	9 vs 4	$p < 0.001$	***
	9 vs 5	$p > 0.05$	n.s.
	9 vs 6	$p < 0.001$	***
	9 vs 7	$p < 0.001$	***
	9 vs 8	$p > 0.05$	n.s.

In the table, different numbers of asterisks denote levels of statistical significance: * indicates $p < 0.05$, ** indicates $p < 0.01$, *** indicates $p < 0.001$, and n.s. indicates $p \geq 0.05$, meaning the result is not statistically significant.

Table S13. The Tukey HSD pairwise comparisons among groups for the flexural modulus of samples after 200 days under different environmental conditions.

Samples	Number	Comparison	p adj	Significance
Before	0	1 vs 0	$p > 0.05$	n.s.
-20℃	1	2 vs 0	$p > 0.05$	n.s.
25℃	2	2 vs 1	$p > 0.05$	n.s.
100℃	3	3 vs 0	$p < 0.001$	***
10%RH	4	3 vs 1	$p = 0.027$	*
50%RH	5	3 vs 2	$p > 0.05$	n.s.
90%RH	6	4 vs 0	$p < 0.001$	***
UV	7	4 vs 1	$p < 0.001$	***
VIS	8	4 vs 2	$p < 0.001$	***
IR	9	4 vs 3	$p < 0.001$	***
/	/	5 vs 0	$p < 0.01$	**
/	/	5 vs 1	$p > 0.05$	n.s.
/	/	5 vs 2	$p > 0.05$	n.s.
/	/	5 vs 3	$p > 0.05$	n.s.
/	/	5 vs 4	$p < 0.001$	***
/	/	6 vs 0	$p < 0.001$	***
/	/	6 vs 1	$p < 0.001$	***
/	/	6 vs 2	$p < 0.001$	***
/	/	6 vs 3	$p < 0.001$	***
/	/	6 vs 4	$p > 0.05$	n.s.
/	/	6 vs 5	$p < 0.001$	***
/	/	7 vs 0	$p < 0.001$	***
/	/	7 vs 1	$p < 0.001$	***
/	/	7 vs 2	$p < 0.001$	***
/	/	7 vs 3	$p < 0.001$	***
		7 vs 4	$p > 0.05$	n.s.
		7 vs 5	$p < 0.001$	***
		7 vs 6	$p > 0.05$	n.s.
		8 vs 0	$p > 0.05$	n.s.
		8 vs 1	$p > 0.05$	n.s.

8 vs 2	$p > 0.05$	n.s.
8 vs 3	$p > 0.05$	n.s.
8 vs 4	$p < 0.001$	***
8 vs 5	$p > 0.05$	n.s.
8 vs 6	$p < 0.001$	***
8 vs 7	$p < 0.001$	***
9 vs 0	$p > 0.05$	n.s.
9 vs 1	$p > 0.05$	n.s.
9 vs 2	$p > 0.05$	n.s.
9 vs 3	$p > 0.05$	n.s.
9 vs 4	$p < 0.001$	***
9 vs 5	$p > 0.05$	n.s.
9 vs 6	$p < 0.001$	***
9 vs 7	$p < 0.001$	***
9 vs 8	$p > 0.05$	n.s.

In the table, different numbers of asterisks denote levels of statistical significance: * indicates $p < 0.05$, ** indicates $p < 0.01$, *** indicates $p < 0.001$, and n.s. indicates $p \geq 0.05$, meaning the result is not statistically significant.

Table S14. The Tukey HSD pairwise comparisons among groups for the ratio of characteristic peak intensities of samples aged at different temperature.

Samples	Number	Comparison	p adj	Significance
Before I_{1730}/I_{1505}	0	1 vs 0	$p < 0.001$	***
-20℃ I_{1730}/I_{1505}	1	2 vs 0	$p < 0.001$	***
25℃ I_{1730}/I_{1505}	2	2 vs 1	$p < 0.001$	***
100℃ I_{1730}/I_{1505}	3	3 vs 0	$p < 0.001$	***
/	/	3 vs 1	$p < 0.001$	***
/	/	3 vs 2	$p < 0.001$	***
Before I_{1460}/I_{1505}	0	1 vs 0	$p < 0.001$	***
-20℃ I_{1460}/I_{1505}	1	2 vs 0	$p < 0.001$	***
25℃ I_{1460}/I_{1505}	2	2 vs 1	$p > 0.05$	n.s.
100℃ I_{1460}/I_{1505}	3	3 vs 0	$p < 0.001$	***
/	/	3 vs 1	$p < 0.001$	***
/	/	3 vs 2	$p < 0.001$	***
Before I_{1370}/I_{1505}	0	1 vs 0	$p > 0.05$	n.s.
-20℃ I_{1370}/I_{1505}	1	2 vs 0	$p > 0.05$	n.s.
25℃ I_{1370}/I_{1505}	2	2 vs 1	$p > 0.05$	n.s.
100℃ I_{1370}/I_{1505}	3	3 vs 0	$p < 0.001$	***
/	/	3 vs 1	$p < 0.001$	***
/	/	3 vs 2	$p < 0.001$	***
Before I_{1060}/I_{1505}	0	1 vs 0	$p > 0.05$	n.s.
-20℃ I_{1060}/I_{1505}	1	2 vs 0	$p < 0.01$	**
25℃ I_{1060}/I_{1505}	2	2 vs 1	$p > 0.05$	n.s.
100℃ I_{1060}/I_{1505}	3	3 vs 0	$p < 0.001$	***
/	/	3 vs 1	$p < 0.001$	***
/	/	3 vs 2	$p < 0.001$	***

In the table, different numbers of asterisks denote levels of statistical significance: * indicates $p < 0.05$, ** indicates $p < 0.01$, *** indicates $p < 0.001$, and n.s. indicates $p \geq 0.05$, meaning the result is not statistically significant.

Table S15. The Tukey HSD pairwise comparisons among groups for the ratio of characteristic peak intensities of samples aged at different relative humidity.

Samples	Number	Comparison	p adj	Significance
Before I_{1730}/I_{1505}	0	1 vs 0	$p < 0.001$	***
10%RH I_{1730}/I_{1505}	1	2 vs 0	$p < 0.001$	***
50%RH I_{1730}/I_{1505}	2	2 vs 1	$p < 0.001$	***
90%RH I_{1730}/I_{1505}	3	3 vs 0	$p < 0.001$	***
/	/	3 vs 1	$p < 0.001$	***
/	/	3 vs 2	$p < 0.001$	***
Before I_{1460}/I_{1505}	0	1 vs 0	$p < 0.001$	***
10%RH I_{1460}/I_{1505}	1	2 vs 0	$p < 0.001$	***
50%RH I_{1460}/I_{1505}	2	2 vs 1	$p < 0.001$	***
90%RH I_{1460}/I_{1505}	3	3 vs 0	$p < 0.001$	***
/	/	3 vs 1	$p < 0.001$	***
/	/	3 vs 2	$p < 0.001$	***
Before I_{1370}/I_{1505}	0	1 vs 0	$p < 0.001$	***
10%RH I_{1370}/I_{1505}	1	2 vs 0	$p > 0.05$	n.s.
50%RH I_{1370}/I_{1505}	2	2 vs 1	$p < 0.001$	***
90%RH I_{1370}/I_{1505}	3	3 vs 0	$p < 0.001$	***
/	/	3 vs 1	$p < 0.001$	***
/	/	3 vs 2	$p < 0.001$	***
Before I_{1060}/I_{1505}	0	1 vs 0	$p < 0.001$	***
10%RH I_{1060}/I_{1505}	1	2 vs 0	$p < 0.001$	***
50%RH I_{1060}/I_{1505}	2	2 vs 1	$p < 0.001$	***
90%RH I_{1060}/I_{1505}	3	3 vs 0	$p < 0.001$	***
/	/	3 vs 1	$p < 0.001$	***
/	/	3 vs 2	$p < 0.001$	***

In the table, different numbers of asterisks denote levels of statistical significance: * indicates $p < 0.05$, ** indicates $p < 0.01$, *** indicates $p < 0.001$, and n.s. indicates $p \geq 0.05$, meaning the result is not statistically significant.

Table S16. The Tukey HSD pairwise comparisons among groups for the ratio of characteristic peak intensities of samples aged at different light radiation.

Samples	Number	Comparison	p adj	Significance
Before I_{1730}/I_{1505}	0	1 vs 0	$p < 0.001$	***
UV I_{1730}/I_{1505}	1	2 vs 0	$p < 0.001$	***
VIS I_{1730}/I_{1505}	2	2 vs 1	$p < 0.001$	***
IR I_{1730}/I_{1505}	3	3 vs 0	$p < 0.001$	***
/	/	3 vs 1	$p < 0.001$	***
/	/	3 vs 2	$p > 0.05$	n.s.
Before I_{1460}/I_{1505}	0	1 vs 0	$p < 0.001$	***
UV I_{1460}/I_{1505}	1	2 vs 0	$p < 0.001$	***
VIS I_{1460}/I_{1505}	2	2 vs 1	$p < 0.001$	***
IR I_{1460}/I_{1505}	3	3 vs 0	$p < 0.001$	***
/	/	3 vs 1	$p < 0.001$	***
/	/	3 vs 2	$p < 0.001$	***
Before I_{1370}/I_{1505}	0	1 vs 0	$p < 0.001$	***
UV I_{1370}/I_{1505}	1	2 vs 0	$p > 0.05$	n.s.
VIS I_{1370}/I_{1505}	2	2 vs 1	$p < 0.001$	***
IR I_{1370}/I_{1505}	3	3 vs 0	$p > 0.05$	n.s.
/	/	3 vs 1	$p < 0.001$	***
/	/	3 vs 2	$p > 0.05$	n.s.
Before I_{1060}/I_{1505}	0	1 vs 0	$p < 0.001$	***
UV I_{1060}/I_{1505}	1	2 vs 0	$p < 0.001$	***
VIS I_{1060}/I_{1505}	2	2 vs 1	$p < 0.001$	***
IR I_{1060}/I_{1505}	3	3 vs 0	$p < 0.001$	***
/	/	3 vs 1	$p < 0.001$	***
/	/	3 vs 2	$p < 0.001$	***

In the table, different numbers of asterisks denote levels of statistical significance: * indicates $p < 0.05$, ** indicates $p < 0.01$, *** indicates $p < 0.001$, and n.s. indicates $p \geq 0.05$, meaning the result is not statistically significant.
